# Positive Correlation of Social Rank and Hair Cortisol Concentration in Group-Housed Pregnant Cows

**DOI:** 10.3390/ani15010013

**Published:** 2024-12-24

**Authors:** Shigeru Ninomiya, Ayumi Nishi, Ririka Nakamura, Mitsuhiro Shibata

**Affiliations:** 1 Faculty of Applied Biological Sciences, Gifu University, Gifu 501-1193, Japan; 2 Graduate School of Natural Science and Technology, Gifu University, Gifu 501-1193, Japan

**Keywords:** animal welfare, avoidance distance, beef cattle, chronic stress, social dominance

## Abstract

In intensive beef production systems, social dominance relationships among cattle and human-cattle relationships constantly affect cattle welfare. However, these factors have not been investigated to assess their long-term effects on cattle welfare. In this study, the relations of hair cortisol concentrations of group-housed pregnant cows with their social rank and avoidance distance when approached by humans were analysed. Thirty-two Japanese black cows were used in this study. Examinations were conducted six times throughout two years, and each cow was subjected to one–three examinations. Hair sampling was conducted on each examination day. The hair cortisol concentrations were measured using a cortisol enzyme immunoassay kit. Social behaviour recordings were made about 6 h on each examination day. The numbers of occurrences of butt, threat, chase, and avoidance behaviours of each animal were recorded using continuous recording methods, and the social rank score for each animal was calculated from the data. Avoidance distances from humans approaching were measured for 23 test cows during the first three examination days. Measurements were taken 5–10 min after the start of morning feeding. The relations between the hair cortisol concentration and social rank score or avoidance distance were analysed using a mixed-effects model or correlation analysis. Significant positive correlation was found between the social rank score and hair cortisol concentration (F_1, 48_ = 4.54, *p* = 0.038). No significant correlation was found between the cows’ responsiveness to humans and their hair cortisol concentration (r = −0.004, *p* = 0.99, *n* = 23). The findings suggest that low-ranking cows had lower cortisol levels.

## Simple Summary

The welfare of group-housed cows, in terms of social dominance and human-cattle relationships, was assessed by analysing hair cortisol. This study found that animals of lower social rank have lower hair cortisol concentrations, and no significant correlation was found between avoidance distance from human and their hair cortisol concentration. The relation between social dominance and animal welfare must be evaluated by various indicators because it includes aspects of animal physical and mental health.

## 1. Introduction

In intensive beef production systems, air quality, high proportions of dietary concentrates, pen floor management, social environments, frequency of inspection, space, and access to feed strongly influence cattle welfare [1]. In group housing, social dominance relationships among cattle resulting from resource competition among individuals affect their welfare [2]. Low social rank individuals are exposed to attack or threat from high social rank animals, often leading to restricted access to feed. Human–cattle relationships can also be included among those factors [3,4]. Increased numbers of animals managed by one stock person in intensive systems may exacerbate the cattle’s fear of humans and this is another factor undermining animal welfare in such systems [4].

The effects of these factors on cattle welfare have been assessed by measuring cortisol concentrations. Cortisol, which is released into the bloodstream from the adrenal cortex by activation of the hypothalamic-pituitary-adrenal (HPA) axis, is an indicator of stress response [5]. Particularly with respect to social dominance relationships, differences in blood cortisol concentrations among social ranks have been reported [6,7]. Regarding human–animal relationships, gentle handling has been found to decrease cattle‘s stress response to handling [3]. Moreover, gentle handling tends to decrease cortisol concentrations in abattoirs [4]. Although these factors affect cattle welfare constantly in housing conditions, they have not been studied to assess their long-term effects on cattle welfare [8]. Measuring hair cortisol concentrations in cattle has been examined and validated as a method to assess long-term stress responses of them [9]. And so, analysing hair cortisol would be suitable for assessing the long-term effects on cattle welfare, compared to sampling blood or faeces, which have been used for assessing short-term or mid-term cortisol levels in cows [5,8].

For this study, hair cortisol concentrations of group-housed cows were measured. Then, their social rank relations and avoidance distances when approached by humans were analysed. It was hypothesised for this study that animals of lower social rank and greater avoidance distance have higher hair cortisol concentrations.

## 2. Materials and Methods

### 2.1. Animals

This study examined 32 Japanese black cows. The animals were housed on the Gifu University farm (Minokamo city, Gifu, Japan). Their mean age when they were examined was 3.78 ± 1.46 years (±SD).

### 2.2. Husbandry Conditions

**Figure 1:**
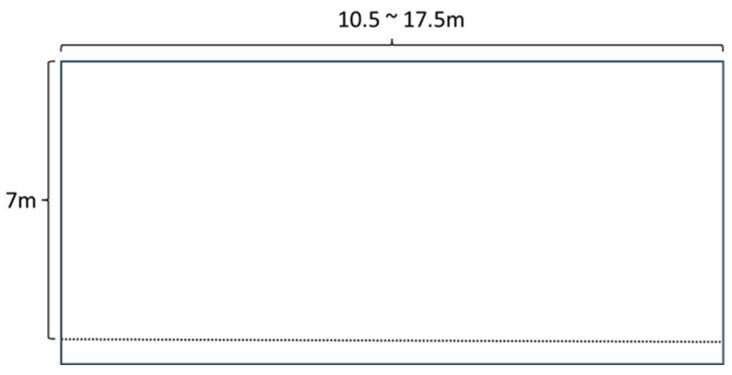
The construction of the examination pen. The dotted line indicated headlocks. The width of the pen differed according to the number of housed cows.

The test cows had been group-housed in one pen with other cows (Figure 1). After pregnancy had been confirmed, cows were transferred to the pen in which they were kept until being transferred to a calving pen before parturition. After parturition, if their pregnancy was confirmed by the next reproduction management, they were transferred to the pen again. Consequently, the number of cows in the pen varied depending on the number of cows confirmed to be pregnant and the date of parturition.

Examinations were conducted 6 times between February 2021 and January 2023. No examination was conducted within two weeks of cows’ introduction into the group or transfer from it. The numbers of cows housed in the pen at the time of examination were 15, 22, 22, 13, 13, and 9, but we examined only cows which were more than thirty days before their estimated parturition date. Therefore, each test cow was subjected to 1–3 examinations. The test cows underwent examinations a median of 103 days (interquartile range 81.25–164.75) before the actual parturition date.

The depth of the pen was fixed at 7 m and its width differed according to the number of cows (10.5–17.5 m, Figure 1), and the mean stocking density (±SD) of the pen at the time of examination was 7.09 ± 1.21 m^2^/cow.

The cows were fed twice daily at 8:30 a.m. and 4:00 p.m. To restrain each animal during feeding, the headlocks installed in front of the feeding troughs (Figure 1) were locked. Water was available ad libitum at all times from a water cup installed in the pen. The housing was naturally ventilated and equipped with fans for heat control.

### 2.3. Examinations

#### 2.3.1. Social Behaviours

Video recordings were taken on each examination day. A handy camera (HDR-CX535/675/680; Sony Corp., Tokyo, Japan) was installed in the examined pen during morning feeding. Video images were recorded from the time of the installation until the start of evening feeding. Based on the video, the numbers of occurrences of butt, threat, chase, and avoidance of each animal were recorded using continuous recording methods (Table 1). The behavioural recording started after headlocks were unlocked after morning feeding, and the recording finished before headlocks were locked before evening feeding for a recording period of about 6 h. A preliminarily study revealed that this arrangement supports the recording of behaviours more than 90 times per animal. One of two observers recorded the behaviours for the first three examinations, and the other recorded them for the last three examinations. Before recording, they confirmed the behavioural definitions, and the second observer used the recorded data of the first observer for training.

The social rank score for each animal was calculated by dividing the total number of butt, threat, and chase behaviours recorded on each observation day by the total number of social behaviours that were recorded [10].

#### 2.3.2. Avoidance Distance Measurement

Avoidance distances when approached by humans were measured for 23 test cows during the first three examination days. Measurements were taken 5–10 min after the start of morning feeding. At the time of measurement, the target animals had already been locked in headlocks, eating hay. The avoidance test was conducted similarly to that used in a study by Windschnurer et al. [11]. The experimenter stood 2 m in front of the target animal. After confirming its attention, the experimenter approached the animal at a speed of one step per second, with the right arm raised 45° in front of the body. At this time, the back of the hand was facing toward the target animal. The experimenter was looking at the tip of the animal’s nose without making eye contact. When the target animal showed a clear avoidance or retraction reaction (side-to-side head movement, attempts to escape from headlocks, or head shaking), the distance between the experimenter’s hand and the tip of the nose of the target animal was measured in steps of 1 cm using a measuring tape. It was defined as the avoidance distance. When the experimenter was able to touch the animal, its cheek was stroked for at least 1 s, but not longer than 3 s. The avoidance distance was recorded as 0 cm. When the animal showed an avoidance or retraction reaction immediately after being touched, the avoidance distance was recorded as 5 cm. After one measurement, the target animal and its three adjacent animals in each side underwent measurements at least 3 min later. Each animal was subjected to two measurements on each examination day. One experimenter performed all measurements.

#### 2.3.3. Hair Sampling and Analysis

Hair sampling was conducted during evening feeding on each examination day using hair from each cow’s tail tuft (switch). Hair at the tip of the tail was collected from the root using a hair clipper (CL-50; Speedik, Osaka, Japan, or ER-GP82-K, Pro Linear Hair Clipper; Panasonic Inc., Osaka, Japan) with an attached blade (2 mm). The collected hair samples were placed in plastic bags and were stored in the dark at room temperature.

A 9 mm length of hair from the root, weighed out to 300–350 mg using an electronic balance (TW223N; Shimadzu Corp., Kyoto, Japan), was used for analysis.

The samples were subsequently washed and dried. After each sample was placed in a 15 mL tube, 5 mL of isopropanol was measured out and added to the tube. Using a multi-shaker (MMS-3000; Tokyo Rikakikai Co. Ltd., Tokyo, Japan), the samples were shaken for 3 min, after which isopropanol was removed. This process (addition of isopropanol, shaking, and removal of isopropanol) was repeated twice for each sample. After the samples were spread on sterile Petri dishes, they were covered with nonwoven paper and were allowed to dry at room temperature.

Each dried hair sample was placed approximately halfway into a tube dedicated to a homogenizer (Beads Crusher µT-01; Taitec Corp., Saitama, Japan). Then, four stainless steel beads (5 mm) were added to the tube. Using the homogenizer, the samples were minced at 4600 rpm for 60 s. This mincing was repeated three times.

The samples were then extracted. After the homogenised sample of each animal was weighed out in two sets of 50 mg using an electronic balance, it was transferred to 1.5 mL tubes. Then, 1 mL of methanol was added to each tube. The samples were incubated for 24 h using a microtube rotator (AS One Corp., Osaka, Japan) tilted at approximately 45°.

The samples were then purified and concentrated. The sample tubes were centrifuged at 12,000 rpm for 2 min at room temperature. For each animal, 0.6 mL of supernatant was taken from each sample tube. Then, 1.2 mL of supernatant, taken from two tubes, was transferred to an empty microtube. Using a block heater (Dry Thermo Unit DTU-1B; Taitec Corp., Saitama, Japan), methanol was evaporated completely in a fume hood at 45 °C for approximately 5 h, with the tube lid open. Subsequently, 0.2 mL of phosphate-buffered saline (pH 7.4) was added to the tube. After each sample was vortexed and reconstituted, it was left to stand overnight in a refrigerator and then stored frozen until use. Before analysis, the frozen samples were thawed at room temperature for approximately 1 h, incubated in a block heater at 50 °C for 5 min, and vortexed.

Analysis was performed using a cortisol enzyme immunoassay kit (Arbor Assays, Ann Arbor, MI, USA). Using a microplate reader (Multiskan FC; Thermo Fisher Scientific Inc., Tokyo, Japan), the absorbance was measured at 450 nm wavelength. Then, the hair cortisol concentration was calculated. The intraassay coefficient of variation was 3.20%.

### 2.4. Data Calculation and Statistical Analyses

For descriptive analysis, the value of social rank score, hair cortisol concentration, and avoidance distance in all examinations of each animal were averaged. Also, their mean value, SE, and interquartile range were calculated.

The relation between the social rank score and hair cortisol concentration was analysed using a mixed-effects model, with the hair cortisol concentration on each examination day as a dependent variable, the social rank score on each examination day as an explanatory variable, and the individual animals as a mixed effect.

The relation between avoidance distance and hair cortisol concentration was examined using correlation analysis. Measured data for each animal were averaged and used for analyses.

Software (JMP Pro 17; JMP Statistical Discovery LLC, Cary, NC, USA, 2022) was used for the analyses.

## 3. Results

The mean values of the social rank score, hair cortisol concentration (pg/mg), and avoidance distance (cm) of the animals were, respectively, 0.572 (SE = 0.036, interquartile range 0.408–0.734), 0.618 (SE = 0.056, interquartile range 0.411–0.760), and 23.7 (SE = 3.2, interquartile range 12.4–31.4).

**Figure 2:**
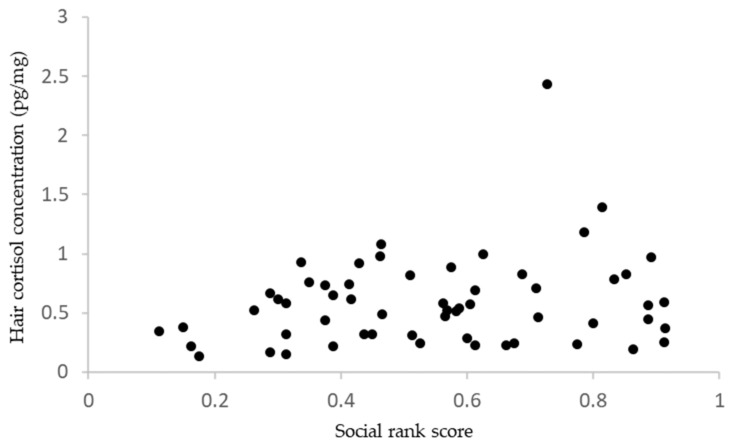
Relationship between social rank score and hair cortisol concentration.

**Figure 3:**
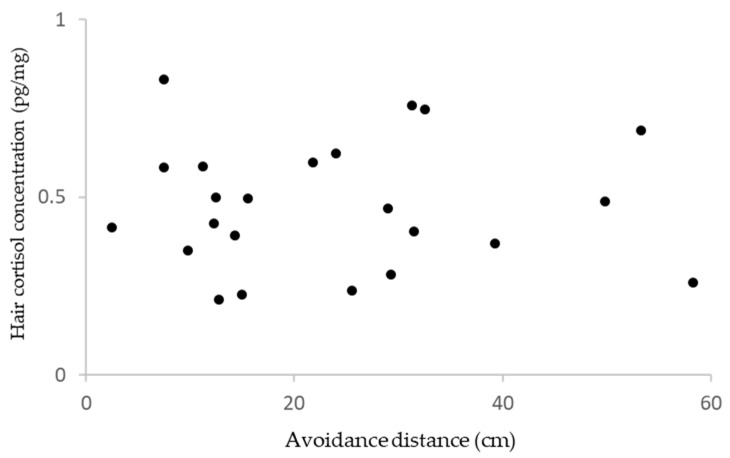
Relationship between avoidance distance and hair cortisol concentration.

Significant positive correlation was found between the social rank score and hair cortisol concentration (coefficient = 0.521, F_1, 48_ = 4.54, *p* = 0.038, Figure 2). No significant correlation was found between avoidance distance and hair cortisol concentration (r = −0.004, *p* = 0.99, n = 23, Figure 3).

## 4. Discussion

### 4.1. Social Rank

In this study, positive correlation was found between social rank scores and hair cortisol concentrations. Hair cortisol concentration is related to the release of endogenous cortisol by HPA axis activity during stimulation [9]. This finding suggests that higher social rank animals have higher activity of the HPA axis, contrary to our hypothesis. Earlier studies investigating the relation between social rank and blood cortisol concentration indicated that high social rank animals have high blood cortisol concentrations [6,7]. Results of earlier studies suggest that high social rank animals have a tendency to challenge stressors in their environment [12]. Presumably, that tendency induced the results. In other earlier studies comparing response to adrenocorticotropic hormone (ACTH) among social ranks [13], high social rank animals showed higher serum cortisol concentrations than either middle or low social rank animals. It was suggested that data obtained under stress condition responses to ACTH of middle and low social rank animals was decreased in the study. Which factor affects the relation between social rank and hair cortisol concentration was not clarified by the results of the present study. Therefore, further studies must be conducted for clarification.

Additionally, some earlier reports [14,15,16,17,18] describe no relation between social rank and blood cortisol concentration. Their results are expected to be affected by confounding factors such as animal attributes (sex, age, breed, etc.), methods used to record social behaviours and social rank scoring, invasiveness of blood sampling (excluding [17]), and the procedures to handle animals before blood sampling. Because social rank indices were all mutually correlated [19], other factors might affect the relation in these reports.

In this study, the test cows were group-housed, and after their pregnancy had been confirmed, they were introduced into the group with which they were kept until being transferred to the calving pen before parturition. Social mixing stimulates cattle’s agonistic behaviours and stimulates the release of endogenous cortisol [20]. Reportedly, physical interactions decrease within three or four days [21,22]. The growth rate of a cow’s switch is 0.58 mm per day [23], and a 9 mm length of hair was used in this study. Therefore, the hair cortisol concentrations reflected the release of endogenous cortisol for about two weeks. Although no examination of social behaviour or hair cortisol was conducted within two weeks of the animal’s introduction into the group or transfer from it in this study, the relation between social dominance and hair cortisol concentrations presumably depends on methods and frequency of social mixing and hair growth rate. Moreover, intensive production system including this study’s housing condition would restrict the function of social behaviours or induce cows’ need for them [24,25], and so the relation between social dominance and hair cortisol concentrations in cows kept at higher apace allowance such as free ranging system might be different from the result of this study.

As one confounding factor, the length of stay in the examined group and pregnancy of cows may be considered. If cows which stay the pen longer were getting closer to their parturition and higher social rank, positive correlation between social rank scores and hair cortisol concentrations might also be induced. However, it was reported in dairy cows that cortisol concentrations measured from blood sampling increase during 240–270 days of cow pregnancy [26], and only one cow in this study was included in the period. Therefore, the probability that the factor affects the relation between social dominance and hair cortisol concentrations seems low.

### 4.2. Avoidance Distance

No relation was found between avoidance distance and hair cortisol concentration. The pregnancy of cows used for this study had already been confirmed. Therefore, no reproduction management or any human-cow interaction was needed, excluding feeding during their stay in the group examined for this study. Because the provision of feed is judged by cattle as a positive interaction with humans [27], it was inferred that the presence or interaction of farm staff would not induce cows’ stress response. A study assessing dairy cows’ stress by measuring faecal cortisol metabolites [8] found no relation between avoidance distance and cortisol concentration. Furthermore, the mean value of avoidance distance in that earlier study was 26.8 cm: the value was identical to that found from this study.

### 4.3. Implication for Animal Welfare

Because assessing animal welfare as positive or negative from cortisol levels alone is impossible [28,29], the findings obtained from this study do not indicate that the welfare of high social rank animals is undermined or that the welfare of low social rank animals presents no difficulty. Animal welfare includes some aspects of animal physical and mental health [30]. Therefore, the relation between social dominance and animal welfare must be evaluated by some indicators originating from multiple perspectives of animal welfare.

## 5. Conclusions

Significant positive correlation was found between the social rank score and hair cortisol concentration, and no significant correlation was found between the cows’ responsiveness to humans and their hair cortisol concentration in this study.

The former results suggest that lower social rank animals have lower hair cortisol concentrations. The reasons of it were thought that higher social rank animals have higher activity of the HPA axis, or responses to ACTH under stress condition differed among social rank animals. Which factor affects the relation between social rank and hair cortisol concentration was not clarified by results of the present study. Therefore, further studies must be conducted for clarification.

## Figures and Tables

**Table 1:** Behaviour definitions.

Behaviour	Definition
butt	cow using the front of her head to make contact with another cow
threat	cow turning toward or approaching another cow with head down and then lunging without making contact
chases	cow actively moving toward another cow, chasing the latter to walk or run away
avoidance	cow actively moving away from another cow whether or not a previous interaction has occurred between the two cows

## Data Availability

Direct request to the corresponding author.
